# Effects of immunogenic cell death-inducing chemotherapeutics on the immune cell activation and tertiary lymphoid structure formation in melanoma

**DOI:** 10.3389/fimmu.2024.1302751

**Published:** 2024-02-07

**Authors:** Hua Zhao, Yu Zhao, Siyuan Zhang, Zhe Wang, Wenwen Yu, Nan Dong, Xuena Yang, Xiying Zhang, Qian Sun, Xishan Hao, Xiubao Ren

**Affiliations:** ^1^ Tianjin Medical University Cancer Institute and Hospital, National Clinical Research Center for Cancer, Tianjin, China; ^2^ Tianjin’s Clinical Research Center for Cancer, Tianjin, China; ^3^ Key Laboratory of Cancer Immunology and Biotherapy, Tianjin, China; ^4^ Haihe Laboratory of Cell Ecosystem, Tianjin, China; ^5^ Department of Immunology, Tianjin Medical University Cancer Institute and Hospital, Tianjin, China; ^6^ Department of Biotherapy, Tianjin Medical University Cancer Institute and Hospital, Tianjin, China

**Keywords:** immunogenic cell death, chemotherapy, immune cell infiltration, high-endothelial venules, tertiary lymphoid structure, PD-1

## Abstract

**Background:**

The infiltration and activation of immune cells in the tumor microenvironment (TIME) affect the prognosis of patients with cancer. Tertiary lymphoid structure (TLS) formation favors tumour- infiltrating-lymphocyte (TIL) recruitment and is regarded as an important indicator of good prognosis associated with immunotherapy in patients with tumors. Chemotherapy is currently one of the most commonly used clinical treatment methods. However, there have been no clear report to explore the effects of different types of chemotherapy on TLS formation in the TIME. This study examined the effects of immunogenic cell death (ICD)-inducing chemotherapeutics on immune cells, high-endothelial venules (HEV), and TLSs in mouse melanomas.

**Methods:**

Doxorubicin (an ICD inducer), gemcitabine (non-ICD inducer), and a combination of the two drugs was delivered intra-peritoneally to B16F1-loaded C57BL/6 mice. The infiltration of immune cells into tumor tissues was evaluated using flow cytometry. HEV and TLS formation was assessed using immunohistochemistry and multiple fluorescent immunohistochemical staining.

**Results:**

Doxorubicin alone, gemcitabine alone, and the two-drug combination all slowed tumor growth, with the combined treatment demonstrating a more pronounced effect. Compared with the control group, the doxorubicin group showed a higher infiltration of CD8^+^ T cells and tissue-resident memory T cells (T_RM_) and an increase in the secretion of interferon-γ, granzyme B, and perforin in CD8^+^ T subsets and activation of B cells and dendritic cells. Doxorubicin alone and in combination with gemcitabine decreased regulatory T cells in the TIME. Moreover, doxorubicin treatment promoted the formation of HEV and TLS. Doxorubicin treatment also upregulated the expression of programmed cell death protein (PD)-1 in CD8^+^ T cells and programmed cell death protein ligand (PD-L)1 in tumor cells.

**Conclusions:**

These results indicate that doxorubicin with an ICD reaction promotes TLS formation and increases PD-1/PD-L1 expression in tumor tissues. The results demonstrate the development of a therapeutic avenue using combined immune checkpoint therapy.

## 1 Introduction

The tumor immune microenvironment (TIME), which comprises abundant immune cells, plays an important role in the antitumor response (1). Tertiary lymphoid structures (TLSs) are organized aggregates of immune cells that form postnatally in non-lymphoid tissues of the TIME (2). The structure is composed of high endothelial venules (HEV) and a variety of immune cells, and is considered the local site where antigen-specific CD8^+^T cells are generated in the tumor microenvironment (3, 4). The presence of TLSs is associated with better prognosis and clinical outcomes in various carcinomas, including non-small cell lung cancer (5, 6), breast cancer (7, 8), ovarian cancer (9, 10), colorectal cancer (11, 12), and melanoma (4, 13) and can serve as a predictive indicator of clinical efficacy in immune checkpoint inhibitor immunotherapy (14, 15). Currently, chemotherapy is still one of the most commonly used methods in clinical treatment, and no studies have explored the effects of different types of chemotherapeutic drugs on TLS in the tumor microenvironment. Hence, it is clinically significant to explore chemotherapeutic drugs that can promote the formation of TLSs to develop a combination approach with immune checkpoint inhibitors.

Chemotherapeutic drugs can be divided into immunogenic cell death (ICD) and non-ICD drugs based on whether they trigger an immune response (16). When ICD occurs, dying cells produce new antigenic epitopes and release damage-associated molecular patterns (DAMPs), such as calreticulin (CRT), high mobility group protein B1 (HMGB1), and adenosine triphosphate (ATP) and then recruit antigen-presenting cells (APCs) to recognize and present antigens on dying cells to T cells (17). A persistent antitumor immune effect is established by activating the immune response system to eliminate the tumor antigens (18). However, whether ICDs induce the formation of tumor-localized HEV and TLSs remains unclear.

As a representative anthracycline drug, doxorubicin mainly acts on DNA to exert cytotoxicity and acts as a representative chemotherapeutic drug for ICD (18). Gemcitabine is a cytidine analog that inhibits DNA synthesis (19). Although related studies have found that gemcitabine can increase CRT exposure and HMGB1 release *in vitro*, it inhibits DAMPs by triggering the action of prostaglandin E2 *in vivo* (20). Therefore, gemcitabine is still considered a non-ICD inducer. In this study, we observed different effects of doxorubicin and gemcitabine on immune cells in the TIME and further discussed the formation of HEV and TLS, which provided a basis for chemotherapy combined with immunotherapy.

## 2 Materials and methods

### 2.1 Cells and culture conditions

Mouse B16-F1 melanoma cells were obtained from the American Type Culture Collection (ATCC). Cells were cultured in RPMI-1640 medium (Cat. L220KJ; Basal Media, Shanghai, China) supplemented with 10% fetal bovine serum (Cat. F801-500; Biocode Biotechnology, Zhejiang, China). The culture conditions involved incubation at 37°C in a humidified incubator containing 5% CO_2_.

### 2.2 *In vivo* mouse tumor model

Female C57BL/6 mice aged between six to eight weeks were purchased from SPF Biotechnology Co. Ltd. (Beijing, China). B16F1 cells were inoculated subcutaneously into C57BL/6 mice at a density of 5×10^5^. Nine days after inoculation, mice were divided into four groups (PBS group, doxorubicin group, gemcitabine group, and combined treatment group) and the corresponding groups were PBS (volume 100ul/mice), doxorubicin (5 mg/kg), gemcitabine (25 mg/kg), and a combination of doxorubicin and gemcitabine for six days, respectively. Doxorubicin (S1208) and gemcitabine (S1149) were purchased from Selleck Chemicals (Houston, TX). After initiating the treatment, tumor length and width were measured daily, and tumor volumes were quantified as (length×width×height)/2. All mice were observed, treated, and euthanized according to the protocols of the Animal Ethics and Welfare Committee of Tianjin Medical University Cancer Institute and Hospital. The animal study protocol was approved by the Animal Ethics and Welfare Committee of Tianjin Medical University Cancer Institute and Hospital (protocol code AE-2021030, September 9, 2021).

### 2.3 Tumor tissue preparation and flow cytometric analysis

The tumor tissues were collected and cut into small pieces of approximately 1 mm^3^. The tumor pieces were incubated with a medium containing DNA hydrolase I and collagenase IV for 30 min at 37°C. A 70-μm filter was used to remove non-digested tissue and obtain a single-cell suspension for the following flow cytometry staining. Cells were first stained with Zombie NIR (Fixable Viability kit, BioLegend) to examine live cells and then surface markers staining with the following antibodies at 4°C for 20 min in the dark: anti-CD45-PE (BioLegend, San Diego, CA, USA), anti-CD3-APC (BioLegend), anti-CD4-FITC (BioLegend), anti-CD19-APC (BioLegend), anti-CD11c-FITC (BioLegend), anti-CD86-PerCP/Cyanine5.5 (BioLegend), anti-PD-1-PE/Cyanine7 (BioLegend), anti-PD-L1-PE/Cyanine7 (BioLegend), anti-CD103- PE/Cyanine7 (BioLegend), anti-PNAd-APC (BioLegend). For intracellular markers, cells were fixed and permeabilized using the FoxP3 staining buffer set (eBioscience) and then stained with intracellular antibodies for 30 min at 4°C, including anti-IFN-γ-PerCP/Cyanine5.5 (BioLegend), anti-Granzyme B-APC (BioLegend), anti-Perforin-PE (BioLegend). Flow cytometry analysis was performed using a BD FACSCanto II flow cytometer (BD Biosciences), and the data were analyzed using FlowJo V10 software.

### 2.4 Immunohistochemistry and multiple immunofluorescence staining

For immunohistochemical analysis, after deparaffinization, rehydration, and antigen repair, tissues were incubated overnight with the primary antibody PNAd (MECA-79, Novus; Shanghai, China). The following day, EIVISON plus (kit-9903, MXB, China) was used for secondary antibody incubation. A DAB kit (ZL1-9019, ZSGB-BIO, China) was used for tissue coloring, hematoxylin was used for nuclear staining, and neutral resin was used to seal the object. Finally, a light-field microscope (Olympus, Tokyo, Japan) was used to examine the stained tumor slices. For multiple immunofluorescence staining, the slides were stained with fluorescently labeled antibodies against CD3 (Abcam, Cambridge, MA, USA), B220 (BioLegend, San Diego, CA, USA), and PNAd (Novus) using a tyramide signal amplification multiplex immunohistochemistry kit (Cat. No. 0004100100; Panovue). According to the immunofluorescence signals, diverse cell types were accurately quantified in each sector of the images using the inForm software (PerkinElmer).

### 2.5 Statistical analyses

All experimental results were statistically analyzed using GraphPad Prism 8. One-way analysis of variance and Dunnett’s test were used to compare the experimental and control groups individually. One-way analysis of variance and Tukey’s test were used for comparisons between any two of the four groups. Values with *P*<0.05 were considered statistically significant.

## 3 Results

### 3.1 Chemotherapy inhibits tumor growth and influences the expression of HMGB1

**Figure 1:**
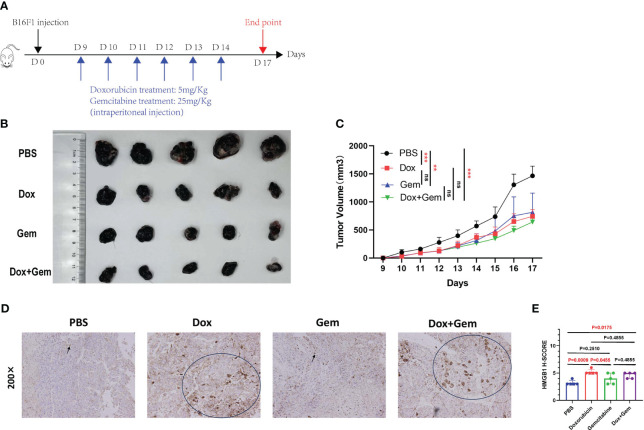
Chemotherapy inhibit tumor growth and affect the expression of HMGB1. **(A)** Schematic diagram of the mice tumor model. First, 5×10^5^ B16F1 cells were inoculated subcutaneously into C57BL/6 mice. Nine days after inoculation, mice were divided into 4 groups (PBS group, doxorubicin group, gemcitabine group, and combined treatment group), and treated with doxorubicin at 5mg/Kg, gemcitabine at 25mg/Kg, and combination with two for 6 days. **(B)** After 17 days, mice were sacrificed and tumors were photographed. **(C)** Tumor length and width were measured every day, and tumor volumes were quantified as (length×width×width)/2. Results were expressed as mean ± SD (n=5), and represented as tumor volume-time curves (**P<0.01, *** *P<*0.001). **(D)** Representative images of HMGB1 immunohistochemical staining in the four groups. **(E)** HMGB1 expression levels were calculated by adding the percentage score of positive staining cells (0-25%, 1 point; 26-50%, 2 points; 51-75%, 3 points; 76-100%, 4 points) and the intensity score (negative staining, 1point; light-yellow, 2 points; yellow-brown, 3 points; dark-brown, 4 points) to calculate the total immunohistochemical score. A statistical bar chart was drawn based on the calculated results.

We established a melanoma mouse model by subcutaneously inoculating B16F1 tumor cells into the groin region of C57BL/6 mice. The detailed administration schedule and tumor harvest intervals are shown in **Figure 1A**. Tumor volumes were monitored daily, the data were plotted (**Figure 1C**), and harvested tumors were photographed (**Figure 1B**).

The results showed that doxorubicin and gemcitabine alone and the combination of both showed significant tumor growth inhibition effects compared to the control group (*P<* 0.001, *P<* 0.01, and *P<* 0.001, respectively). The combination treatment group showed the most significant inhibitory effect, but no statistically significant difference was noted than that in the other two groups (**Figure 1C**).

HMGB1 (formerly known as HMG-1), a highly conserved ubiquitous protein, has been described as a nuclear DNA-binding protein involved in nucleosome stabilization and gene transcription. The release of HMGB1 from the nucleus into the surroundings of the dying cells is an important characteristic of ICD (17). Immunohistochemical staining was used to evaluate the cytoplasmic expression of HMGB1 in tumor tissues after different treatments. The results showed that doxorubicin treatment demonstrated an increase in the expression of HMGB1 compared to the control and gemcitabine treatments (5.2% ± 0.45% vs. 3.2% ± 0.45%, *P*=0.0009, 5.2% ± 0.45% vs. 4.0% ± 1.00%, *P*=0.0455). In addition, the expression of HMGB1 was also significantly upregulated in the combination treatment group than in the control group (4.6% ± 0.55% vs. 3.2% ± 0.45%,*P*=0.0175) (**Figures 1D, E**).

### 3.2 Effects of doxorubicin on lymphocyte subpopulation

**Figure 2:**
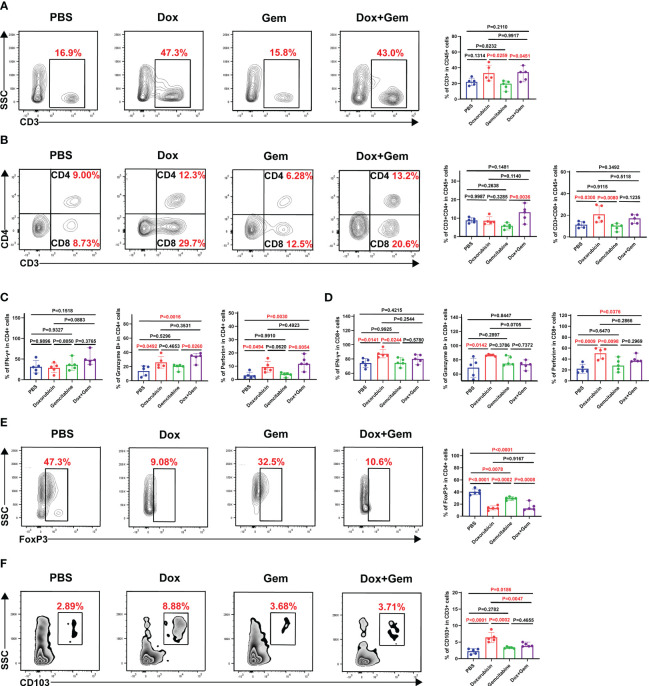
Effects of doxorubicin on T cell Subsets. Mice with melanoma were treated with doxorubicin, gemcitabine, and combination of both, and tumors were harvested to produce a single-cell suspension at day 17, which was analyzed by flow cytometry. **(A)** Representative plots of CD3^+^ cells gated on CD45^+^ cell population, and histogram of percentage of CD3^+^ cells in CD45^+^ cells from the four groups (n=5). **(B)** Representative plots of CD4^+^ cells, CD8^+^ cells gated on CD3^+^ cell population, and histogram of percentage of CD4^+^ cells and CD8^+^ cells in CD3^+^ cells from the four groups (n=5). **(C, D)** Histogram of positive percentage of IFN-γ, granzyme B and perforin in CD4 and CD8 cells, respectively. **(E)** Representative dot plot of Foxp3^+^ cells within the CD4^+^ gate and histogram of percentage of Foxp3^+^ cells in CD4^+^ cells from the four groups (n=5). **(F)** Representative dot plot of CD103^+^ cells within the CD3^+^ gate and histogram of percentage of CD103^+^ cells in CD3^+^ cells from the four groups (n=5).

To study the changes in the immune microenvironment after doxorubicin treatment, single-cell suspensions were prepared from tumor tissues for flow cytometry analysis. The results showed that the proportion of CD3^+^ T cells in doxorubicin group and combination treatment group was significantly higher than that in gemcitabine group (33.2% ± 10.1% vs. 18.1% ± 5.54%, *P=* 0.0259; 31.9% ± 8.40% vs. 18.1% ± 5.54%, *P=* 0.0451) (**Figure 2A**). Doxorubicin alone can significantly promote infiltration of CD8^+^ T cells compared to control and gemcitabine treatments (20.9% ± 7.77% vs. 11.4% ± 2.85%, *P=* 0.0300; 20.9% ± 7.77 vs. 9.33% ± 3.02%, *P=* 0.0080); however, it did not exhibit a significant effect on CD4^+^ T cells (**Figure 2B**). Next, we analyzed the levels of cytotoxic cytokines secreted by CD4^+^ T cells and CD8^+^ T cells. Compared with the control treatment, both doxorubicin alone and combination treatment promoted the expression of granzyme B (25.9% ± 8.27% vs. 13.9% ± 7.13%, *P* = 0.0492; 33.1% ± 6.39% vs. 13.9% ± 7.13%, *P* = 0.0061) and perforin in CD4^+^ T cells (9.45% ± 4.16% vs. 3.13% ± 2.35%, *P* = 0.0494; 12.7% ± 5.0% vs. 3.13% ± 2.35%, *P* = 0.0030). However, the results showed no significant effect on IFN-γ expression (**Figure 2C**). In CD8^+^T lymphocytes, compared with the control treatment, doxorubicin increased the expression of IFN-γ (87.5% ± 5.75% vs. 74.4% ± 6.15%, *P*= 0.0141), granzyme B (86.5% ± 1.30% vs. 68.9% ± 13.3%, *P*= 0.0142), and perforin respectively. (50.5% ± 10.4% vs. 22.2% ± 8.09%, *P=* 0.0009; **Figure 2D**).

Foxp3-expressing regulatory T (Treg) cells suppress effective tumor immunity and are associated with poor prognosis in patients with cancer (21). In this study, the proportion of Tregs in all treatment groups was significantly decreased compared than that in the control group (doxorubicin group:13.6% ± 3.38% vs. 40.2% ± 4.84%,*P*< 0.0001; gemcitabine group: 29.4% ± 2.49% vs. 40.2% ± 4.84%,*P*= 0.0078; combined group:15.5% ± 6.29% vs. 40.2% ± 4.84%,*P*< 0.0001; **Figure 2E**).

Furthermore, tissue-resident memory T cells (T_RM_), which are important components of tumor infiltrating lymphocytes (TILs) were also evaluated in this study. T_RM_ cells mediate anti-tumor immunity by producing cytolytic mediators and releasing cytokines and chemokines to recruit and activate immune cells (22). Compared with the control treatment, doxorubicin alone and the combination treatment significantly promoted the infiltration of CD103^+^ T_RM_ cells (6.43% ± 1.48% vs. 2.24% ± 0.80%, *P*< 0.0001; 4.15% ± 0.56% vs.2.24% ± 0.80%, *P*= 0.0186) (**Figure 2F**).

### 3.3 Doxorubicin promoted recruitment and activation of antigen-presenting cells

**Figure 3:**
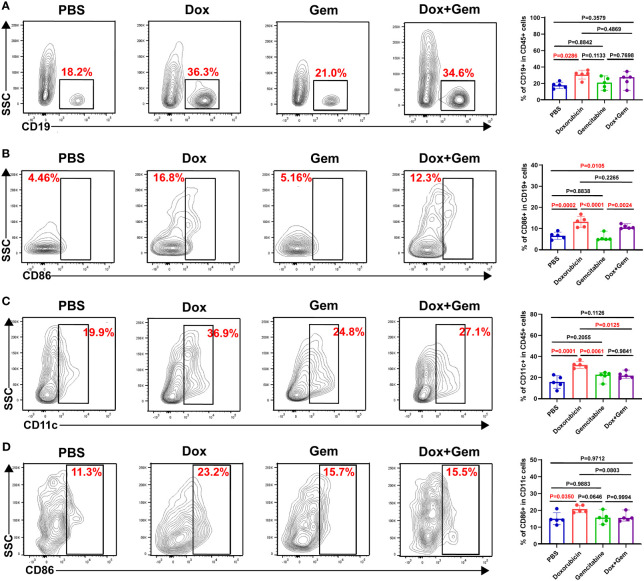
Doxorubicin promoted antigen-presenting cells recruitment and activation. B cells, and DCs were gated by CD19, and CD11c respectively. The activated cells were labeled with CD86. **(A)** Representative dot plot of CD19^+^ cells within the CD45^+^ gate and histogram of percentage of CD19^+^ cells in CD45^+^ cells from the four groups (n=5). **(B)** Representative dot plot of CD86^+^ cells within the CD19^+^ gate and histogram of percentage of CD86^+^ cells in CD19^+^ cells from the four groups (n=5). **(C)** Representative dot plot of CD11c^+^ cells within the CD45^+^ gate and histogram of percentage of CD11c^+^ cells in CD45^+^ cells from the four groups (n=5). **(D)** Representative dot plot of CD86^+^ cells within the CD11c^+^ gate and histogram of percentage of CD86^+^ cells in CD11c^+^ cells from the four groups (n=5).

APCs play key roles in the initiation and regulation of innate and adaptive anti-tumor immune responses (23, 24). In the present study, compared with the control treatment, doxorubicin treatment promoted the recruitment of B cells (30.7% ± 5.46% vs. 17.8% ± 3.84%, *P*= 0.0286) and dendritic cells(DCs) (31.9% ± 3.23% vs. 15.7% ± 5.89%, *P*= 0.0001) to the tumor tissue site (**Figures 3A, C**). Furthermore, both the doxorubicin alone and the combination treatment increased the proportion of CD86^+^ B cells compared to the control treatment (13.2% ± 2.60% vs. 6.57% ± 1.69%, *P*= 0.0002; 10.8% ± 0.94% vs.6.57% ± 1.69%, *P*= 0.0105). Gemcitabine treatment did not increase the infiltration or activation of B cells (*P* > 0.05; **Figure 3B**). The data showed that only doxorubicin group showed increased proportion of CD11c^+^DC and CD86^+^DCs compared to the control group (20.8% ± 2.18% vs.15.1% ± 3.57%, *P*= 0.0350; **Figure 3D**).

### 3.4 Doxorubicin promotes HEV and TLS formation

**Figure 4:**
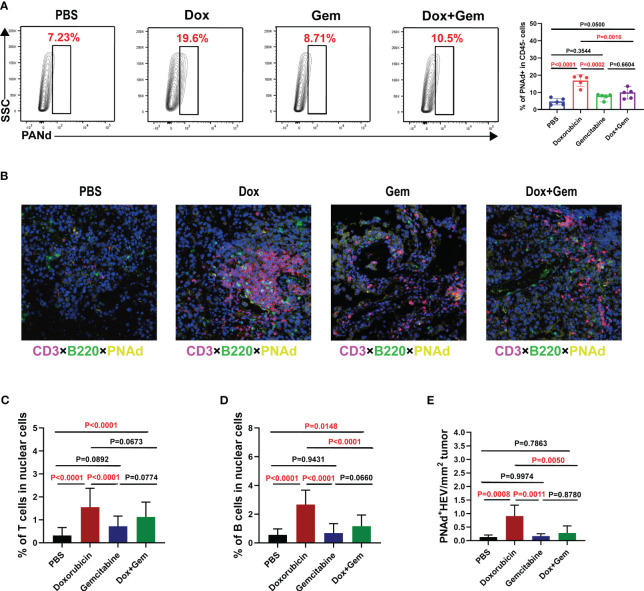
Doxorubicin promotes HEV and TLS formation. **(A)** Representative dot plot of PNAd^+^ cells within the CD45^-^ gate and histogram of percentage of PNAd^+^ cells in CD45^-^ cells from the four groups (n=5). **(B)** Representative images of multiple immunofluorescence staining (magnification, ×200) in control group, doxorubicin group, gemcitabine group, and combination treatment group, respectively. The slide was stained with CD3 (red), B220 (green), PNAd (yellow), and DAPI (blue). **(C-E)** Five fields were randomly selected for each sample, and the proportion of T and B cells occupying nuclear cells was quantified using inForm software (PerkinElmer) based on immunofluorescence signals. **(E)** The density of PNAd^+^ HEV was used to quantify TLS formation.

The HEV is the main portal for lymphocytes entering the tumor tissues and is the most important component of TLSs (25). We measured the proportion of PNAd^+^ HEV in CD45^-^ cells by flow cytometry. The proportion of PNAd^+^ HEV in the doxorubicin group was significantly higher than that in the control, gemcitabine, and combination treatment groups (*P<* 0.0001, *P*= 0.0002, and *P*= 0.0016, respectively; **Figure 4A**).

Multiple immunofluorescence staining was used to evaluate the formation of TLSs (**Figure 4B**). Five fields were randomly selected from multiple immunofluorescence-stained samples, and the proportion of T and B cells was quantified using the inForm software. The proportion of CD3^+^ T cells was significantly increased in the doxorubicin group than in the control and gemcitabine groups (1.55% ± 0.83% vs. 0.31% ± 0.35%, *P<* 0.0001; 1.55% ± 0.83% vs. 0.31% ± 0.35%, *P<* 0.0001, **Figure 4C**). Moreover, the combination treatment group showed similar increase in T-cell infiltration compared with the control group (1.13% ± 0.65% vs.0.31% ± 0.35%, *P<* 0.0001, **Figure 4C**). The proportion of B220^+^B cells was significantly increased in the doxorubicin group than in the control, gemcitabine and combination treatment groups (2.67% ± 1.01% vs.0.55% ± 0.42%,*P<* 0.0001; 2.67% ± 1.01% vs.0.68% ± 0.67%,*P<* 0.0001; 2.67% ± 1.01% vs.1.16% ± 0.79%,*P<* 0.0001, **Figure 4D**). We also measured the density of PNAd^+^ HEV in the samples to quantify the TLS formation. The results showed that doxorubicin treatment significantly promoted TLS formation compared with the control, gemcitabine, and combination treatment (0.91% ± 0.40% vs.0.14% ± 0.07%, *P*= 0.0008; 0.91% ± 0.40% vs.0.17% ± 0.09%, *P*= 0.0011; 0.91% ± 0.40% vs.0.28% ± 0.26%, *P*= 0.0050, **Figure 4E**). These data are consistent with the flow cytometry results.

### 3.5 Doxorubicin upregulates PD-1/PD-L1 expression

**Figure 5:**
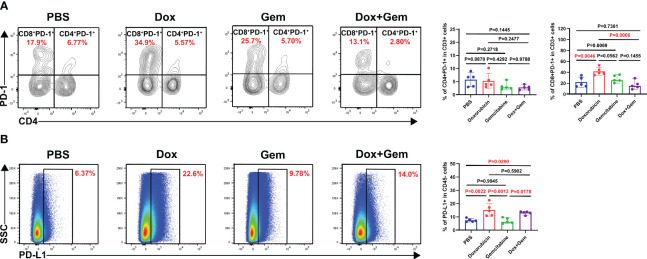
Doxorubicin upregulate PD-1/PD-L1 checkpoint expression. **(A)** Representative dot plot of PD-1^+^ cells within the CD4 and CD8 gates and histogram of percentage of PD-1^+^ cells in CD4^+^ and CD8^+^ cells from the four groups (n=5). **(B)** Representative dot plot of PD-L1^+^ cells within the CD45- gate and histogram of percentage of PD-L1^+^ cells in CD45^-^ cells from the four groups (n=5).

Finally, we analyzed the changes in PD-1 expression in CD4^+^ and CD8^+^ T cells after different treatments. The flow cytometry results showed that doxorubicin significantly increased the expression of PD-1 on CD8^+^ T cells compared with the control and combination treatments (41.9% ± 7.17% vs.22.1% ± 9.27%, *P*= 0.0046; 41.9% ± 7.17% vs.17.1% ± 7.66%, *P*= 0.0006, **Figure 5A**). No statistically significant differences were noted in the expression PD-1 in CD4^+^ T cells among the four groups.

Next, we observed the expression of PD-L1 on CD45^-^ tumor cells. The results showed that the doxorubicin significantly increased the PD-L1 expression compared with the control and the gemcitabine treatments (15.3% ± 4.83% vs.7.53% ± 1.29%,*P*= 0.0022; 15.3% ± 4.83% vs. 7.10% ± 2.06%,*P*= 0.0013, **Figure 5B**). The combination treatment group also showed increased proportion of PD-L1 on tumor cells compared with the control and the gemcitabine group (13.0% ± 1.26% vs.7.53% ± 1.29%,*P*= 0.0290;13.0% ± 1.26% vs.7.10% ± 2.06%,*P*= 0.0178, **Figure 5B**). These findings indicate that combining doxorubicin treatment with anti-PD1 immunotherapy may yield potential benefits.

## 4 Discussion

The immune system plays a crucial role in the elimination of tumors. In the TIME, TLSs, which include B-cells- and T-cell-enriched areas, may be the local site of initiation and maintenance of humoral and cellular immune responses against cancers (26, 27). Numerous studies have evaluated the formation of TLSs in different cancers and their association with patient prognosis (5–13). Moreover, the presence of TLS in the TIME is an important indicator of the effectiveness of immune checkpoint therapy (28). Therefore, exploring strategies that can induce TLS formation is crucial for proposing combination therapies to improve the efficacy of immune checkpoint therapy.

ICD induction in tumor cells is a promising approach for activating anti-tumor immune responses (16). Chemotherapeutics that can promote ICD can recruit and promote DC maturation and cross-initiation of tumor-specific CD8^+^ T cells via DAMP release (29, 30). HMGB1 plays a crucial role in this process. Via binding to toll-like receptor 4 (TLR-4) on DCs, HMGB1 promotes DC maturation and releases pro-inflammatory cytokines to trigger an effective immune response (31). Another study showed that HMGB1, synergistically with ATP, could induce DCs to release interleukin‐1β (IL-1β), and HMGB1-specific antibodies can block the ability of IL-1β production in DCs after exposure to dying tumor cells (32). In the present study, doxorubicin as a representative chemotherapeutics of ICD was used as the study. Doxorubicin treatment promoted the infiltration and activation of DCs and enhanced the function of infiltrated CD8^+^ T cells, such as the expression of IFN-γ, granzyme B and perforin, which is consistent with the previous reports (33).

The role of B cells in cancer immunity and their implications in new immunotherapies have garnered significant interest. B cell function includes not only antibody secretion but also antigen presentation to T cells. In human cancers, antigen-presenting B cells are defined as a subset of CD86^+^CD21^-^ B cells (34). CD86^+^ B cells colocalize with T cells in TLSs and are enriched in tumors with increased numbers of TLSs (35). Compared to CD8^+^ T cells alone, the co-localization of B cells with CD8^+^ T cells increases patient survival (14, 36). One study showed that oncolytic viruses acting as ICD inducers can increase the expression of CD86 in B cells; however, it is unclear whether this is related to ICD (37). In this study, the flow cytometry data showed a significant increase in the total number of B and CD86^+^ B cells after doxorubicin treatment.

Furthermore, the present results showed that doxorubicin treatment can significantly increase the proportion of CD103^+^ T_RM_. They can conveniently perform local immune monitoring functions in tumors and exhibit tumor-specific immune protective effects. CD103^+^ T_RM_ cells can directly kill epithelial-derived tumor cells by producing a large number of cytotoxic particles and cytokines, such as granzyme-B, perforin, and IFN-γ (38). T_RM_ cells may also produce chemokines, leading to the recruitment of XCR1^+^BATF3^+^DC and activation of T cells (39). These results further indicate that chemotherapeutics with the capacity of ICD induction might be more beneficial for combination immunotherapy.

PNAd is a hallmark of HEV that facilitates the recruitment of lymphocytes to lymphoid organs and serves as a marker of TLS formation (40). DCs promote the maturation of HEV endothelial cells via lymphotoxin-β, which plays an important role in monitoring the entry of lymphocytes into TLS (41, 42). Whether chemotherapeutics with an ICD induction impact the formation of TLS remains unclear. In the present study, we compared the levels of HEV formation between doxorubicin and gemcitabine and found that doxorubicin induced high PNAd expression and promoted TLS formation. The increased proportion and maturity of DCs in the doxorubicin treatment group confirmed the positive effects of doxorubicin on TLS formation. This may be the link between the onset of ICD and TLS induction.

Additionally, PD-1 expression in CD8^+^ T cells and PD-L1 expression in tumor cells was elevated after doxorubicin treatment. The high expression of PD-L1 on tumor cells in the TIME may be related to the inflammatory signals, such as IFN-γ, generated during the anti-tumor immune response (43). Our results verify the aforementioned conclusions and provide a theoretical basis for combined anti-PD-1/PD-L1 therapy. In addition, some chemotherapeutics inhibit Treg production with ICD (44–46), which is consistent with our results. This suggests that therapeutics mediating ICD reaction can disrupt the immunosuppressive state of the tumor microenvironment.

Although our experiment showed that doxorubicin promoted the infiltration of immune cells and the formation of TLS simultaneously, this study still has some limitations. First, only one ICD inducer was used. Secondly, the results were examined only using a mouse model of melanoma. Therefore, additional chemotherapeutics with ICD reactions and additional experimental models should be used for further confirmation. In conclusion, the results elucidated that doxorubicin, with an ICD reaction, promoted TLS formation and increased PD-1/PD-L1 expression in tumor tissues, which may be advantageous for combined immune checkpoint therapy.

## Data availability statement

The original contributions presented in the study are included in the article/supplementary material. Further inquiries can be directed to the corresponding authors.

## Ethics statement

The animal study was approved by the Animal Ethics and Welfare Committee of Tianjin Medical University Cancer Institute and Hospital. The study was conducted in accordance with the local legislation and institutional requirements.

## Author contributions

HZ: Project administration, Supervision, Writing – review & editing, Data curation, Writing – original draft. YZ: Data curation, Project administration, Writing – original draft, Writing – review & editing, Methodology, Validation. SZ: Data curation, Writing – review & editing. ZW: Writing – review & editing. WY: Writing – review & editing. ND: Writing – review & editing. XY: Writing – review & editing. XZ: Writing – review & editing. XH: Writing – review & editing, Funding acquisition, Project administration, Supervision. XR: Writing – review & editing, Funding acquisition, Project administration, Supervision. QS: Writing – review & editing.
